# Identification and characterization of planarian multinucleated cells in *Schmidtea mediterranea* using imaging flow cytometry

**DOI:** 10.3389/fcell.2025.1611516

**Published:** 2025-11-28

**Authors:** Annasha Dutta, Anna Samelak-Czajka, Magdalena Trybus, Marcin Osuch, Anastasiia Zaremba, Paulina Jackowiak

**Affiliations:** 1 Laboratory of Single Cell Analyses, Institute of Bioorganic Chemistry Polish Academy of Sciences, Poznań, Poland; 2 Department of Molecular and Systems Biology, Institute of Bioorganic Chemistry Polish Academy of Sciences, Poznań, Poland

**Keywords:** imaging flow cytometry, FACS, multinucleated cells, planarian regeneration, progenitor cells

## Abstract

Cellular polyploidy plays critical roles in both pathological and physiological processes. While extensively studied in organisms like *Drosophila* and zebrafish, and mammalian tissues such as the liver, kidney and heart, the existence of multinucleated polyploid cells in planarians, particularly asexual species, remains poorly understood. Here, we describe a novel approach combining imaging flow cytometry and fluorescence-activated cell sorting, to identify and characterize a stable population of presumed multinucleated cells in *Schmidtea mediterranea* under both regenerating and non-regenerating conditions. Insights into nuclearity, along with cell and nuclear size, suggest that these cells are formed through mechanisms involving cell fusion and/or endomitosis. Furthermore, analysis of marker gene expression patterns in the population containing multinucleated cells, upon neoblast depletion, suggests that they are likely undifferentiated. In addition, knockdown of the late epidermal progenitor marker *Agat-1* followed by AGAT-1 custom antibody-based staining of cell populations indicates that the majority of MuNs are possibly late epidermal progenitor cells. Overall, the new findings presented here pave the way for further exploration into the biological significance of multinucleated cells in planarian regeneration and tissue maintenance.

## Introduction

Cellular polyploidy is a widespread phenomenon observed across metazoans. It refers to particular cells in diploid organisms that contain multiple sets of chromosomes in the nucleus or are multinucleated (Peterson and Fox, 2021). Polyploidy is extensively linked to both pathological conditions, such as cancer and foreign body responses, and physiological processes. For example, in mammals, polyploid cells play essential roles in early embryonic development, as well as in regeneration of heart, liver, eye, kidney and bladder (Lazzeri et al., 2018; Wilkinson et al., 2019; Anatskaya and Vinogradov, 2022). Similarly, other organisms representing evolutionary distant phyla, like zebrafish and *Drosophila*, have adopted comparable strategies using polyploid cells for tissue maintenance and restoration (Cao et al., 2017; Anatskaya and Vinogradov, 2022). Additionally, these cells are also involved in resilience to genotoxic insults, exemplified by irradiation-resistant bone marrow multinucleated cells that aid regeneration (Moein et al., 2023).

Polyploid cells can form through both cell cycle-dependent and independent pathways. In the latter scenario, cells fuse to create a syncytium, as in the case of muscle development (Richardson et al., 2008). The cell cycle-dependent mechanism is a more common pathway of polyploid cell formation, referred to as endoreplication, which can be further divided into endocycle and endomitosis. During endocycle, the cell bypasses the mitosis (M) phase, which results in the formation of a polyploid mononucleated cell with replicated DNA and an enlarged nucleus (Cohen et al., 2018). In contrast, endomitosis involves mitosis followed by failed cytokinesis, hence increasing cellular ploidy by the formation of multinucleated cells. This pathway is common in liver, heart and bladder cells for development, repair and regeneration (González-Rosa et al., 2018; Wang et al., 2018; Donne et al., 2020). Notably, endocycle and endomitosis often do not operate in isolation; they can occur simultaneously or in a sequential manner (Bailey et al., 2021).

Freshwater planarian species have served as models in regeneration research due to their remarkable ability to regenerate entire organisms, including the nervous system, from small body fragments. This capability is attributed to a heterogeneous population of stem cells, called neoblasts, constantly present in an adult organism (Rink, 2013; Reddien, 2018). It is therefore not surprising that neoblasts have been the primary research focus, when studying planarian regeneration. However, the phenomenon of polyploidy in planarians and its potential relevance for regeneration has received very little attention thus far. Consequently, polyploidization in the form of multinucleated cells in these animals has been documented only in limited instances. Formation of multinucleated cells has been observed in the embryonic development of the sexual planarian, *Schmidtea polychroa* (Cardona et al., 2005). In asexual planarian, *Schmidtea mediterranea*, sigma neoblasts were shown to undergo abnormal multinucleation via endoreplication, as a result of inhibition of the condensin I complex crucial for chromosomal segregation (Lai et al., 2018). To date, nonetheless, there have been no reports about the existence of polyploid multinucleated cells in asexual planarians under homeostatic conditions or during regeneration.

Flow cytometry and fluorescence-activated cell sorting (FACS) have been the preferred methods for studying and isolating various planarian cell populations. Due to the unavailability of antibodies against cell-specific marker proteins, such cell populations have been segregated based on cell size and nuclear content. Accordingly, a landmark study described a FACS method utilizing triple fluorescent staining—propidium iodide for dead cell exclusion, Hoechst 33342 for nuclear staining, and Calcein AM for cytoplasmic staining—to distinguish planarian cell populations with varied differentiative potentials, viz., X1 (neoblasts or stem cells), X2 (progenitors), and Xins (differentiated cells) (Hayashi et al., 2006). Since then, this approach has become the gold-standard and has been widely used in advanced studies, including the creation of the first planarian cell-type atlas via single-cell transcriptomics (Plass et al., 2018). Multiple variations of the original technique have been developed, using other dyes, excitation lasers and sorting options, to enable enhanced subpopulation profiling and increase viability of sorted cells (Hayashi et al., 2010; Peiris et al., 2016; Hayashi and Agata, 2018; Kuroki and Agata, 2023; Wang et al., 2024; Zhang et al., 2024). Despite relying on nuclear content for the identification of cell populations, none of these studies directly investigated the presence of polyploid cells. This is reasonable, as distinguishing cells with a stably increased chromosome number from those that are mitotically active and synthesizing DNA, presents a significant challenge, particularly when examining a highly heterogeneous cell suspension derived from the dissociation of an entire organism. Moreover, further differentiation of polyploid cells into those containing a single polyploid nucleus and those that are multinucleated is beyond what conventional flow cytometry can achieve.

Imaging flow cytometry (IFC) is a multiparametric technique that combines high throughput of classical flow cytometry with spatial information provided by microscopy. Thus, it offers unbiased measurements of fluorescent signal intensities for tens of thousands of cells per sample, while providing precise information on the signal localization within individual cells. IFC platform using Draq5 or Hoechst 33342 nuclear staining is increasingly being used to study multinucleated cells in the context of cancer (Rodrigues et al., 2014; Rodrigues, 2018; Vorobjev et al., 2023).

In this study, we present a novel approach to identify and characterize a rare but stable population of wild-type planarian presumed multinucleated cells under both regenerating and non-regenerating conditions. Cells of the asexual strain of *S. mediterranea* were analyzed using IFC and the traditional FACS platforms. The analysis of morphological features observed in IFC-generated images of multinucleated cells allowed us to propose a potential mechanism of their formation. In summary, we introduce a method to explore a previously uncharacterized planarian cell subset that appears to be undifferentiated late epidermal progenitors. We believe that future studies on these cells shall further expand our understanding of the planarian regeneration process.

## Materials and methods

### Animal husbandry and RNAi

Asexual strain of *S. mediterranea* was maintained as previously described (Newmark and Sánchez Alvarado, 2000). For the experiments requiring regenerating fragments, 20 wild-type animals were cut into three fragments–head, trunk and tail – and equal numbers of fragments were collected at 0, 24, 72 and 120 h post amputation (hpa) and analysed further.

RNAi mediated gene knockdown experiments were separately performed by the introduction of artificial dsRNAs synthesized against *H2B* and *Agat-1* respectively, using AmpliScribe T7-Flash Transcription Kit (Epicentre #ASF3507), via microinjections into the worms. A total of 15 animals unfed for a week were injected with 32 nL of 2 μg/μL dsRNA, using Nanoject III™ (Drummond Scientific Company) according to the following regime: injection for 3 consecutive days, a gap of 2 days and, again injection for next 2 consecutive days, for a total of 5 days. All injected worms were collected at 5 days post injection (dpi) and prepared alongside control worms for downstream analysis. Control samples were from the same number of wild-type worms without any injections. All experiments were performed in 3 independent biological replicates.

Primer sequences are provided in Supplementary Table S1 (S1).

### Cell dissociation and staining

A total of 20–30 whole worms were suspended in ice-cold CMFB (Calcium Magnesium-Free Buffer with 1% BSA) and dissociated into single-cells using the gentleMACS™ Octo Dissociator (Miltenyi Biotec). Each sample was subjected to the spleen-1 dissociating program (55 s pulse) for 3 times to achieve homogenous single-cell suspension with minimal debris. The dissociated cells were filtered through 50 µm CellTrics™ filters and centrifuged at 400 rcf and 4 °C to collect the cell pellet. Supernatant was discarded, leaving 50 µL. First, live cell cytoplasm was stained with 0.4 μg/mL Calcein AM dye (ThermoFisher Scientifc # 65-0853–78; Ex/Em 495/515 nm) in 500 µL CMFB solution, followed by a step of PBS wash. Wherever applicable, 1× CellBrite® Steady 550 (Biotium #30107-T; Ex/Em 550/570 nm) in PBS was then added to the cell pellet to stain the cell membrane. The dye was prepared and used according to the manufacturer’s instructions. After a 30-min incubation, no wash step was performed before proceeding to nuclear staining. Subsequently, nuclear staining was performed using 10 µM Draq5 (ThermoFisher Scientific #62251; Ex/Em 646/697 nm). At this stage, the cells were not washed and were directly proceeded for flow cytometry analysis.

To investigate whether MuNs produce AGAT-1 protein, a marker of late epidermal progenitors, a custom anti-AGAT-1 antibody conjugated with Alexa Fluor 488 (Ex/Em 490/525 nm) was used. This antibody was generously provided by Genotic. The antibody was designed using an AI-driven approach, based on the protein sequence predicted from SMEST051964001.1 transcript, obtained in an animal-free system, and has not been fully validated. Cells were fixed using IC Fixation Buffer (eBioscience #00-8222-49) and permeabilized with 1x Permeabilization Buffer (eBioscience #00-8333-56). Following permeabilization, around 1 × 10^6^ cells were stained with 2 µg of the anti-AGAT-1-Alexa Fluor 488 antibody, washed with 1x Permeabilization Buffer and stained with 10 µM Draq5 (ThermoFisher Scientific #62251) as described above.

### RNA isolation, cDNA synthesis and PCR amplification

#### RNA isolation, cDNA synthesis and PCR amplification from whole worms

15-20 worms were flash frozen in liquid nitrogen. Total RNA was isolated using mirVana™ miRNA Isolation Kit, with phenol (ThermoFisher Scientific #AM1560). Total RNA samples were treated with TURBO™ DNase (ThermoFisher Scientific # AM2238) and DNA-free RNA samples were purified using standard ethanol-sodium acetate precipitation method. The purified RNA sample concentrations were measured on Qubit 4 Fluorometer (Invitrogen) using Qubit™ RNA Broad Range (BR) Assay Kit (Invitrogen, #Q10210).

cDNA was synthesised from 1 µg of total RNA sample using random hexamer primers and SuperScript™ IV Reverse Transcriptase (ThermoFisher Scientifc #18090010). For samples that required PCR amplification of a target to be used as template for subsequent dsRNA synthesis, primers containing T7 promoter sequence (5′ TAATACGACTCACTATAGGG 3′) were used. The amplification reactions were carried out using Herculase II Fusion DNA Polymerase (Agilent Technologies #600677).

#### RNA isolation and cDNA synthesis from sorted cells

Total RNA was isolated and treated with DNase using the Total RNA Zol-Out™ D (A&A Biotechnology, #043-100) and dissolved in 20 µL water. Subsequently, 10 µL of the isolated total RNA was proceeded for cDNA synthesis using the High-Capacity cDNA Reverse Transcription kit with RNase Inhibitor (Applied Biosystems, #4374966).

### Quantitative RT-PCR approaches

#### qRT-PCR for obtaining individual marker gene expression proportions within sorted samples

qRT-PCR primers were designed for *Smedwi1, Agat-1* and *GST-1* genes. The amplification reactions were performed in 3 technical replicates for each cDNA sample using 5x HOT FIREPol®EvaGreen® qPCR Supermix (Cytogen #08-36-000001). Analysis was done using 2^−ΔCt^ method. ΔCt values were normalized with respect to expression of reference gene *H.55.12e*.

#### qRT-PCR for comparing gene expression profiles between H2B knockdown (H2B KD)/Agat-1 knockdown (Agat-1 KD) and WT samples

qRT-PCR primers were designed for *Smedwi1, NB21.11e, H2B, Agat-1, PC2* and *GST-1* genes. The amplification reactions were performed in 3 technical replicates for each cDNA sample using 5x HOT FIREPol® EvaGreen® qPCR Supermix (Cytogen #08-36-000001). All comparisons were made between *H2B* KD/*Agat-1* KD and WT samples. Analysis was done using 2^−ΔΔCt^ method. ΔCt values were normalized with respect to expression of reference gene *Ef2* and fold change values were calculated.

Primer sequences are provided in Supplementary Table S1.

### Imaging flow cytometry

Imaging flow cytometry was performed on the Cytek® Amnis® ImageStream®X Mk II cytometer equipped with two CCD camera detectors (Cytek Biosciences, USA). INSPIRE software (Cytek Biosciences) was used for data acquisition. The images were captured using a 40× objective at a low fluidics speed. Brightfield images were obtained in channels 1 and 9, side scatter (SSC) images were obtained in channel 6 (745–785 nm filter), using a 785 nm laser with power of 3.75 mW; Calcein AM and Alexa Fluor 488 conjugated to anti-AGAT-1 antibody were detected in channel 2 (480–560 nm filter) using a 488 nm laser with power of 4 mW and 120 mW, respectively; CellBrite Steady 550 was detected in channel 3 (560–595 nm filter) using a 561 nm laser power of 20 mW; Draq5 was detected in channel 11 (642–745 nm filter) using a 642 nm laser with power of 30 mW and, Hoechst 33342 was detected in channel 7 (435–505 nm filter) using a 405 nm laser ×40 with power of 20 mW. At least 60,000 events were acquired from each unsorted sample and a minimum of 2,000 events from each sorted sample. For AGAT-1-oriented experiments, a minimum of 5,000 events per sample were acquired. IDEAS v6.2 software (Cytek Biosciences) was used for data analysis. The analysis strategy has been described in relevant sections of the Results. For the measurement of cell and nuclear diameters, approximately 50–200 cells from each category, MuNs and MoNs (within individual cell populations), were analyzed from a representative dataset.

### Fluorescence-activated cell sorting (FACS)

FACS was performed with a high-speed flow cytometer BD FACSAria™ Fusion (Becton Dickinson). Calcein AM was excited with 488 nm laser and detected in FITC channel (BP 530/30). Draq5 was illuminated using 640 nm laser and detected in APC channel (BP 670/30). The cells were sorted using 100 μm nozzle and 4-way purity sorting mode. Approximately 50,000 cells were sorted per sample into Eppendorf® Protein LoBind 1.5 mL tubes, containing 300 µL PBS with 1% BSA for downstream imaging flow cytometry, or 350 µL TRIzol™ Reagent (Invitrogen, #15596018) for total RNA extraction. The former was processed immediately, and the latter were vigorously shaken once out of the sorter and kept on ice until the next step. Data were analyzed using FACSDiva 9.0.1 software (Becton Dickinson).

### Statistical analysis

The experiments were made in three replicates, unless explicitly stated otherwise. For comparisons involving more than two groups or involving two or more groups, but exhibiting zero variance in any condition, statistical analyses were performed using one-way ANOVA followed by Tukey’s *post hoc* test. To account for multiple testing, the Holm–Bonferroni method was applied for p-value adjustment. For comparisons between two groups, a two-sample two-sided t-test was conducted with Holm–Bonferroni adjustment. A threshold of significance was set at p < 0.05 for all analyses.

## Results

### Identification of planarian multinucleated cells using flow cytometry

**FIGURE 1:**
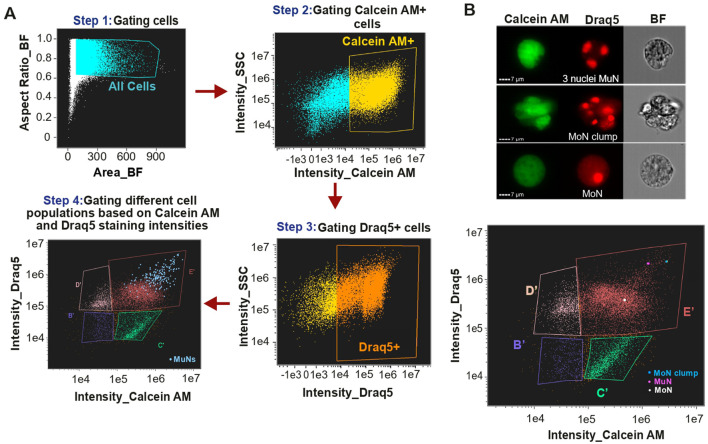
**(A)** Detailed scheme for identifying multinucleated cells on Cytek® Amnis® ImageStream®X Mk II (IS) using IDEAS® data analysis software. **(B)** Images depicting selected MuN, MoN and MoN clumps followed by a plot showing the localization of these specific cells within MuNs containing population E’.

Given the widespread occurrence of polyploid cells in metazoans, we hypothesized that such cells are also present in *S. mediterranea*. Thus, we undertook an exploratory study in search of multinucleated cells (MuNs), as their polyploid nature can be more definitively established compared to mononucleated cells (MoNs), for which ploidy assessment is more prone to errors. First, we aimed at devising a stepwise gating strategy to identify true MuNs on the IFC Platform, Cytek® Amnis® ImageStream®X Mk II (IS). The cells were stained with Calcein AM and Draq5, where Calcein AM stains cytoplasm of viable cells and Draq5 stains nuclei of live or fixed cells upon DNA binding. Nuclei-containing viable Calcein AM and Draq5 double positive cells were sequentially gated using unstained sample as control (Figure 1A, Steps 1-3). Next, these double positive cells were visualized on Calcein AM vs. Draq5 intensity plot, which allowed us to identify four distinct populations, denoted as B′, C′, D′ and E′, hereafter referred to as IS populations (Figure 1A, Step 4). Preliminary visual assessment of cell images on the Draq5 channel revealed the existence of cells with more than one Draq5 positive spots, considered to be MuNs. Majority of them were found in population E′, with only a few (∼3–4) present in the adjacent population D′. Characterized by large cells with high intensities of both Calcein AM and Draq5 (Figure 1A, blue dots on the dot plot from Step 4), population E′ primarily consisted of large mononucleated cells (MoNs) and a small fraction of MoN clumps. To accurately distinguish multinucleated cells (MuNs) from these other objects, we performed a detailed visual inspection of all objects within gates D′ and E′, identifying the rare MuNs, at a frequency of ∼2% in E′ population (Supplementary Table S2). This translated to a frequency of ∼1–2% in all cells double positive for Calcein AM and Draq5 (Calcein^+^Draq5^+^). This analysis compared images from three channels, brightfield (BF), Calcein AM and Draq5. While both MuNs and MoN clumps on the Draq5 channel displayed multiple nuclear spots, images from the BF and Calcein AM channels distinguished MuNs from MoNs clustered together (Figure 1B).

To address concerns that MuNs might be artifacts of Draq5 staining, we analyzed cell suspensions stained with an alternative nuclear dye, Hoechst 33342. MuNs were indeed observed in similar proportions (Supplementary Figure S1) as in Draq5-stained samples. However, the population containing MuNs in Hoechst 33342-stained samples was less distinct compared to Draq5-stained samples. Consequently, all subsequent studies were conducted using Calcein AM and Draq5 staining for greater clarity and accuracy. Overall, we identified presumed MuNs in wild-type planarians, exploiting the IFC platform. The Calcein AM and Draq5 staining intensity values were amongst the highest for these cells, within the total double positive cell pool.

### Morphological characterization of the MuNs using imaging flow cytometry

**FIGURE 2:**
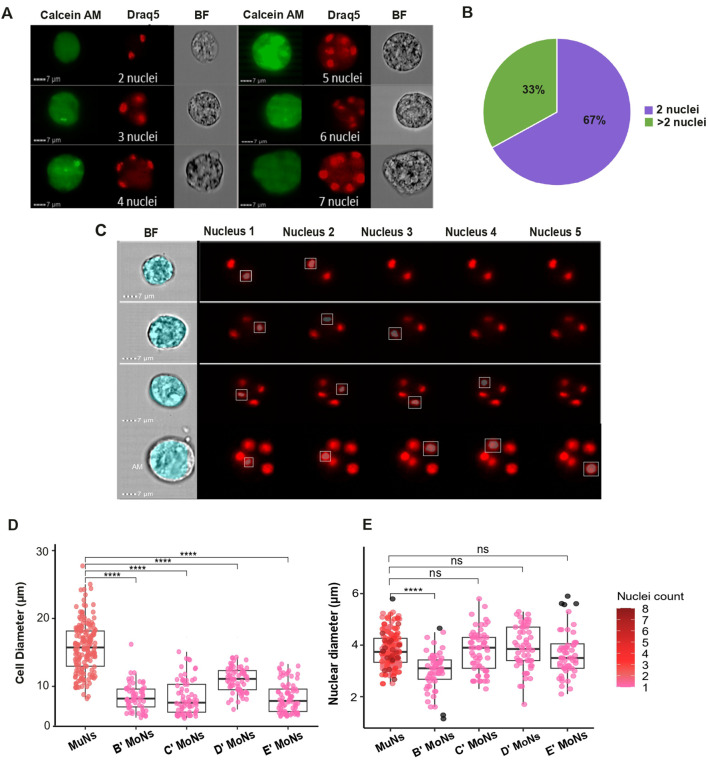
**(A)** Exemplar image panel showing variation of nuclearity amongst planarian MuNs. Scale:7 μm; magnification: 40×. **(B)** Chart showing the general composition of planarian MuN cell pool, with respect to the number of nuclei. **(C)** Images from IDEAS software showing custom BF and Draq5 channel masks created to optimally cover the BF cell image and Draq5 nuclear images. **(D)** Cell diameters of MuNs and MoNs representing four IS cell populations. Statistical analyses were performed using one-way ANOVA followed by Tukey’s *post hoc* method (*p-value: 0.05*). **(E)** Nuclear diameters of MuNs and MoNs representing four IS cell populations. The color gradient represents the number of nuclei in a particular cell. Statistical analyses were performed using one-way ANOVA followed by Tukey’s post hoc method (p-value: 0.05).

The size and nuclearity of multinucleated cells vary widely (Brodbeck and Anderson, 2009; Vorobjev et al., 2023). Therefore, to morphologically characterize planarian MuNs we analyzed the cell images based on three criteria: nuclearity (the number of individual nuclei within the cell), cell diameter and nuclear diameter. The number of nuclei within the multinucleated cell pool varied between individual cells. Specifically, the planarian MuNs could be broadly grouped into two categories: 2-nucleated (2nuc) and >2-nucleated (>2nuc). Accordingly, while most MuNs had 2 nuclei (∼67%), a stable percentage was >2 nucleated (∼33%), ranging from 3 to 8 nuclei per cell (Figures 2A,B).

Further, to measure the cellular and nuclear diameters of MoNs and MuNs, we created custom BF and Draq5 image masks to optimally cover the cell and nuclear surface areas, respectively. To obtain cell diameter values, the BF mask was adjusted using the erode function in a way that it correctly fits the BF channel cell images. The nuclear diameters of individual nuclei of each multinucleated cell were measured using the combination of three IDEAS masks: LevelSet, Watershed and Component on Draq5 channel nuclear image. For setting the optimal nuclear mask, three main aspects were considered: (i) to cover a particular nuclear spot area adequately, (ii) to ignore stain halos extending beyond the actual spot area and (iii) to differentiate between two closely placed spots as two different nuclei. Component masks are capable of identifying user-defined objects (in this case, individual nuclei stained with Draq5) one at a time. When combined, the three masks (Component, LevelSet and Watershed) enable the accurate and separate analysis of one or more nuclei within a cell, distinguishing both diffusely stained and distinctly stained nuclei as separate nuclear entities within MuNs. To achieve optimal nuclear identification, individual Component masks (defining nuclear identity) were paired with user-defined constant values for LevelSet and Watershed masks (ensuring accurate nuclear coverage). This approach was designed to accommodate the maximum number of nuclei observed in a planarian MuN, which in this case was 8. Images depicting example masks correctly covering cell and nuclear areas are shown in Figure 2C. The images displaying nuclear masks include five Component masks (combined with LevelSet and Watershed masks), each corresponding to individual nuclei within MuNs containing 2–5 nuclei. In cells with less than 5 nuclei, such as a 2-nucleated MuN – specific nuclear diameter values were recorded for nuclei 1 and 2, while the values from component masks 3, 4, and 5 were zero. This demonstrated the accuracy of the mask development strategy. Using this strategy, we observed that although MuNs had significantly bigger diameters than MoNs, ∼16 µm vs. ∼8–10 μm, respectively (Figure 2D), the nuclear diameters for all cells were generally constant at ∼3–4 µm (Figure 2E) (Supplementary Table S2). These parameters, cell and nuclear diameters, served as indirect measurements for their respective sizes. Overall, MuNs could be described as larger cells containing 2-8 nuclei per cell and having comparable nuclear sizes as that of MoNs. The fact that the nuclear sizes of these 2 cell subsets were similar supported the notion that MuNs indeed contained several nuclei, rather than a single nucleus with multiple foci.

**FIGURE 3:**
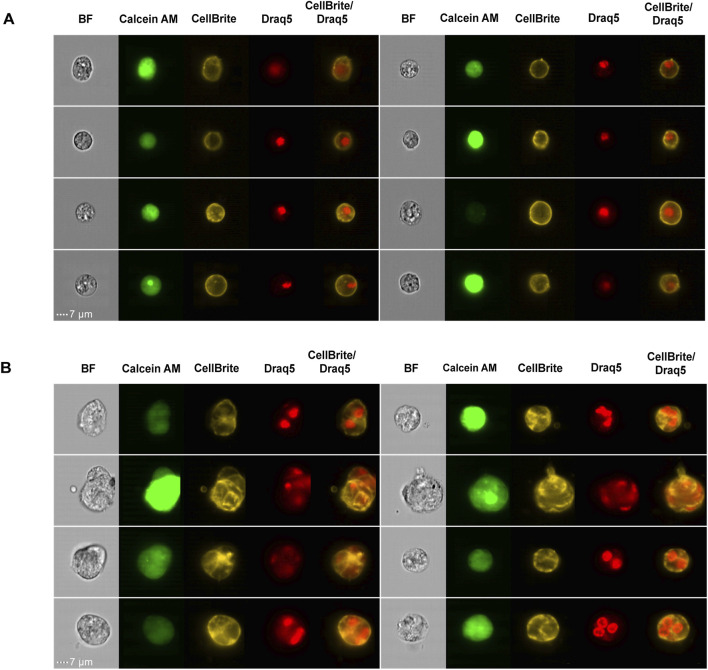
**(A)** Picture gallery of representative planarian MoNs, with nuclear and membrane staining. **(B)** Picture gallery of representative planarian MuNs, with nuclear and membrane staining. Scale:7 μm; magnification: 40×.

To further characterize the morphological features of MuNs, we visualized the cell membranes using CellBrite, a dye that binds to membrane proteins. We observed that CellBrite efficiently labeled the plasma membrane without significantly penetrating intracellular membranes (Figure 3A). As a result, each cell appeared surrounded by a clearly defined membrane signal, providing a distinct outline of individual cell borders. Interestingly, in some MuNs we observed structures that differed from typical single-cell morphology. These MuNs appeared as clusters of contiguous cells, but their appearance was clearly distinct from random clumps generated during tissue dissociation. In these cases, a single MuN was composed of two or more tightly connected cells forming a coherent structure. This unique membrane staining pattern revealed that individual nuclei of such MuNs were separated from each other via plasma membranes, indicating internal compartmentalization within the larger structure (Figure 3B). The morphology observed here, lacking regular patterns, was interpreted as a late stage of cell fusion, rather than cytokinesis. These findings suggested that, at least in some cases, MuNs biogenesis involves a cell fusion mechanism.

### Marker gene expression profiling of sorted cell populations

**FIGURE 4:**
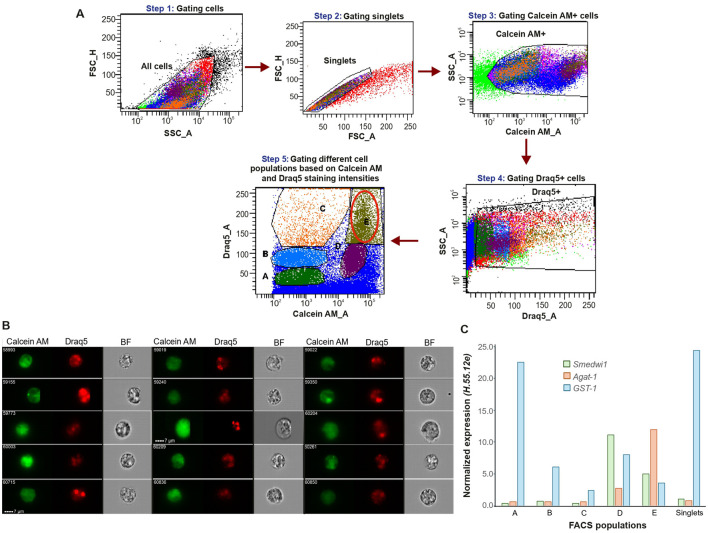
**(A)** Specific population gating strategy for sorting of dissociated planarian cells. **(B)** Picture gallery of sorted cells from population E containing MuNs. **(C)** Expression of selected marker genes in each sorted sample, represented as 2^-ΔCt^ values normalized to the reference gene H.*55.12e*. Median values from 2 replicates are presented.

Our next objective was to isolate the cell population containing MuNs for molecular characterization. For this, Calcein AM and Draq5 stained cells were analyzed using FACSAria™ Fusion cell sorter and FACSDiva™ 9.0.1 software as follows. Cells were gated out from the debris, followed by doublet discrimination (Figure 4A, Steps 1 and 2, respectively). After identifying the Calcein AM- and Draq5-positive cells (Figure 4A, Steps 3-4), the double positive cells were plotted on a Calcein AM vs. Draq5 intensity plot (Figure 4A, Step 5). Clearly, due to the intrinsic instrument-related differences, the obtained dot plot presented the acquired events in a distinct way than previously seen in the case of the imaging cytometer. Accordingly, here we gated 5 cell populations, denoted A-E, hereafter referred to as FACS populations (Figure 4A, Step 5). Based on the high staining intensities of both dyes, population E was postulated to contain the MuNs.

To verify this assumption, we visualized the cells from each of the FACS gates. To this end, ∼50,000 cells from each population were sorted and immediately visualized separately on IS. Other than MoNs, we found multiple MuNs within population E, as expected (Figure 4B). MuNs constituted ∼2% of population E, similar to what was observed for population E′ of non-sorted IS samples. This result confirmed our hypothesis that population E contains MuNs and that cell sorting does not alter their distribution within population E. By inspecting the other sorted populations on IS individually, we additionally confirmed that these populations were comprised of MoNs of varying sizes, having different staining intensities for Calcein AM and Draq5 (Supplementary Figure S2). Lastly, we wanted to ensure that MuNs, which are larger than MoNs, were not being substantially excluded by the singlet gate, originally set to remove larger entities such as doublets and/or clumps. Accordingly, we skipped the step of gating singlets (Figure 4A, Step 2) while preserving all the downstream steps to eventually obtain double positive cells. We named the new population E as E1, whose position on the Calcein AM vs. Draq5 dot plot was identical to that of the original population E. Using IS, we observed that sorted population E1 also contained a similar number of MuNs with respect to other cells in the population. Indeed, few larger MuNs were obtained, but most of them were similar in size and morphology to those obtained from population E (Supplementary Figure S3).

To molecularly characterize all the five FACS populations, we again sorted cells and performed qPCR profiling of selected marker genes. A sorted sample of singlets, containing a mix of cells from all targeted populations and untargeted regions of the dot plot, was used as a proxy for the suspension of all cell types. The selected marker genes represented three classes of cells at various stages of differentiation – *Smedwi1* (neoblast); *Agat-1* (late epidermal progenitor) and *Glutathione S transferase-1* or *GST-1* (differentiated phagocyte - dd_Smed_v6_20_0_1). As all of the sorted samples were heterogenous mixtures of cells, the relative expression level of each marker in a particular sample reflected the approximate proportion of the corresponding cell type within that population. Accordingly, populations A, B, and C majorly consisted of differentiated cells. Population D was likely dominated by neoblasts, inferred from the highest proportion of the neoblast marker expression. Population E exhibited the highest proportion of the late epidermal progenitor marker gene expression compared to all other populations, suggesting a predominance of progenitor cell states. Additionally, populations D and E showed the lowest expression of the differentiated cell marker, further supporting their classification as undifferentiated cell populations (Figure 4C). However, it is important to note that none of these individually sorted populations represented homogenous cell types. Altogether, despite the heterogeneity within each population, the undifferentiated marker gene expression signatures suggested that the MuN-containing population E likely represents undifferentiated subtypes.

### MuNs are stably maintained throughout the course of planarian regeneration

**FIGURE 5:**
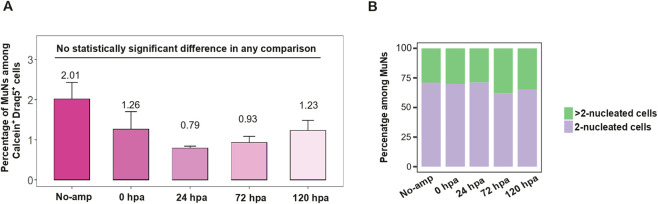
**(A)** The percentage of MuNs in the pool of all Calcein^+^Draq5^+^ cells for worms at various regenerative time points and non-regenerating (No-amp) worms. Statistical analyses were performed using one-way ANOVA followed by Tukey’s *post hoc* method (*p-value: 0.05*) - median values from 3 replicates are presented. **(B)** Distribution of 2-nuc vs. > 2-nuc MuNs within the MuN pool of worms at various regenerative time points and non-regenerating (No-amp) worms. Median values from 3 replicates are presented.

Given the possible undifferentiated state of MuNs, we next studied them along a defined time frame of regeneration. To this end, planarians were cut, and cell dissociates were analyzed using IS at 0, 24, 72 and 120 h post amputation (hpa). A dissociate of non-amputated worms was used as control. The percentage of MuNs in the pool of all Calcein^+^Draq5^+^ cells fluctuated between various regenerative time points and non-amputated worms, but the changes did not reach statistical significance. This means that irrespective of the regenerative state, the total proportion of MuNs was stable (Figure 5A). To analyze the MuNs further, we calculated the proportions of 2nuc and >2nuc cells in all the above conditions. We found that there was no significant difference in proportions between the samples (Figure 5B). The consistent presence of MuNs in both regenerative and non-regenerative life stages suggest they are a consistent component of the basal homeostatic state of planarians.

### Limited reduction of MuNs following neoblast depletion is indicative of their progenitor cell nature

From the qPCR analysis of sorted cell populations, we had initial indication suggesting that planarian MuNs could be undifferentiated. To further investigate this, we aimed to examine the effects of targeted neoblast depletion on MuNs and the MuN-containing cell population.

**FIGURE 6:**
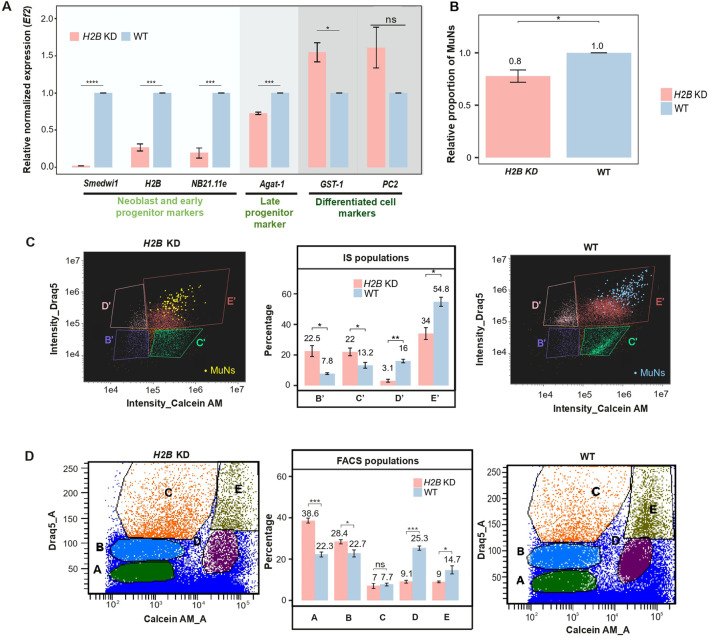
**(A)** Marker gene expression levels in *H2B* KD and WT samples. Expression levels were normalized to the reference gene (*Ef2*) and represented relative to WT (set as 1). Statistical analyses were performed using one-way ANOVA followed by Tukey’s *post hoc* method (*p-value: 0.05*) - median values from 3 replicates are presented. **(B)** The proportion of MuNs in *H2B* KD samples relative to WT (set as 1). Statistical analyses were performed using a two-sided t-test (*p-value: 0.05*) – median values from 3 replicates are presented. **(C)** Comparison of IS population distribution between *H2B* KD and WT samples. Yellow and blue dots represent manually assigned MuNs. Statistical analyses were performed using one-way ANOVA followed by Tukey’s *post hoc* method (*p-value: 0.05*) – median values from 3 replicates are presented. **(D)** Comparison of FACS population distribution between *H2B* KD and WT samples. Statistical analyses were performed using one-way ANOVA followed by Tukey’s *post hoc* method (*p-value: 0.05*) – median values from 3 replicates are presented.

Neoblast depletion was achieved by knocking down the *H2B* gene (*H2B* KD) using RNAi. At first, gene expression analyses were conducted on *H2B* KD vs. wild-type (WT) worms, focusing on three groups of marker genes representing neoblasts, progenitors, and differentiated cells. Neoblasts and early progenitors were marked by *Smedwi1* and *NB21.11e* (SMEST024707001.1), respectively, late progenitors by *Agat-1*, and differentiated cells by the phagocytic marker *GST-1* and the neural marker *prohormone convertase-2* (*PC2*, dd_Smed_v6_1566_0_1). To this end, we used qPCR to analyze marker gene expression in *H2B* KD worms relative to WT. As expected, we observed a sharp decrease in the expression of neoblast and early progenitor markers. Notably, there was also a significant reduction in the expression of the late epidermal progenitor marker. These changes were accompanied by an increase in the expression of differentiated cell markers, reflecting a compensatory enrichment in the number of differentiated cells due to the absence of neoblasts and progenitors in the constant cell pool (Figure 6A).

To check if *H2B* KD directly affected the MuNs, we next calculated the percentage of these cells under RNAi vs. WT conditions. To this end, we analyzed cell dissociates for both sample types using IS. We found that the proportion of MuNs was marginally reduced in the *H2B* KD samples compared to WT, reaching approximately 0.8 relative to the WT level (normalized to 1). (Figure 6B). This led us to further analyze all the individual populations identified earlier using IS and FACS. Based on the IS plots, we found that there were two populations whose percentage contributions to the total pool of all Calcein^+^Draq5^+^ cells significantly decreased as a result of *H2B* KD. Percentage of population D′ in *H2B* KD was 0.2 times the WT value (3.1% in *H2B* KD and 16% in WT) and for E′ containing MuNs, the percentage in *H2B* KD was 0.6 times the WT value (34% in *H2B* KD and 54.8% in WT). This helped us conclude that these two populations contained undifferentiated cell types and based on the levels of reduction of the two, it was concluded that population D′ contained mostly neoblasts and the lesser affected population E′ was majorly comprised of progenitors at various stages (Figure 6C). As for the FACS populations, we again obtained two populations whose percentage contributions to the total cell pool were particularly reduced. Population percentages of E (containing the MuNs) and D were 0.4 times (9.1% in *H2B* KD and 25.3% in WT) and 0.6 times (9% in *H2B* KD and 14.7% in WT) of their respective WT values (Figure 6D). As the reduction in population D was much more profound than that of E, it could be concluded that D majorly contains neoblasts and E, progenitors. This was in line with the observations presented above (Figure 6A) that the progenitor marker expression was reduced to a much lesser extent than the neoblast marker, and populations D and E have the highest expressions of the neoblast and progenitor markers (Figure 4C), respectively.

### Knockdown of the late epidermal progenitor marker gene *Agat-1* causes pronounced MuNs depletion

**FIGURE 7:**
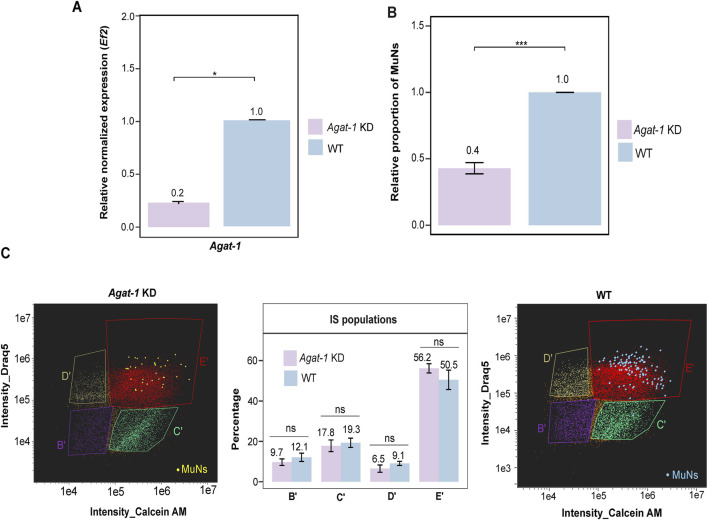
**(A)** *Agat-1* gene expression level in *Agat-1* KD and WT samples. Expression level was normalized to the reference gene (*Ef2*) and represented relative to WT (set as 1). Statistical analyses were performed using one-way ANOVA followed by Tukey’s *post hoc* method (p-value: 0.05) – median values from 3 replicates are presented. **(B)** The proportion of MuNs in *Agat-1* KD samples, relative to WT (set as 1). Statistical analyses were performed using a two-sided t-test (p-value: 0.05) - median values from 3 replicates are presented. **(C)** Comparison of IS population distribution between *Agat-1* KD and WT samples. Yellow and blue dots represent manually assigned MuNs. Statistical analyses were performed using one-way ANOVA followed by Tukey’s *post hoc* method (p-value: 0.05) - median values from 3 replicates are presented.

Given that the MuNs-containing population E appeared to represent progenitor cells, we examined how the proportion of MuNs would be affected upon the knockdown of *Agat-1*. After verification of the knockdown efficiency (Figure 7A), we analyzed cell dissociates from *Agat-1* KD and WT worms using IS, and quantified MuNs in both sample types. We observed a marked reduction in the abundance of MuNs in *Agat-1* KD samples compared to WT, reaching approximately 0.4 relative to the WT level (normalized to 1) (Figure 7B). This was clearly more pronounced than the reduction seen upon *H2B* depletion. We then analyzed particular cell populations, this time focusing solely on the IS-based classification. Notably, no significant changes were observed in the percentages of any cell population, including population E’ (Figure 7C). These results indicated that *Agat-1* knockdown exerted a preferential effect on MuNs, without broadly affecting the composition of major cell populations.

### Majority of the MuNs are positive for AGAT-1, a marker of late epidermal progenitors

**FIGURE 8:**
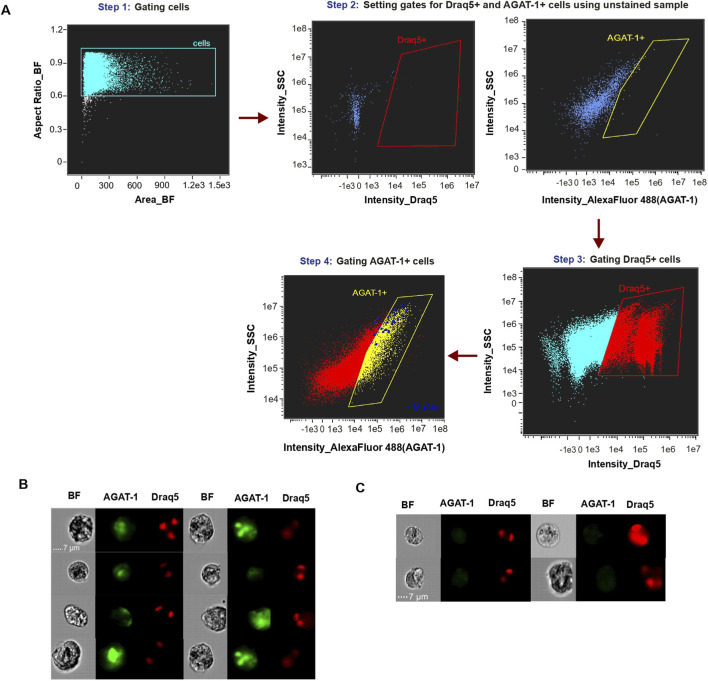
**(A)** Gating strategy of AGAT-1 positive cells on Cytek® Amnis® ImageStream®X Mk II (IS) using IDEAS® data analysis software. MuNs are indicated as blue dots, and those located within the yellow “AGAT-1+” gate represent MuNs positive for the AGAT-1 protein. **(B)** Picture gallery of AGAT-1 positive MuNs. **(C)** Picture gallery of AGAT-1 negative MuNs. Scale:7 μm; magnification: 40×.

The observed reduction in MuNs numbers following *Agat-1* knockdown implied that AGAT-1 is important for the formation or maintenance of MuNs. However, this result did not directly address whether MuNs themselves produce the AGAT-1 protein. To investigate this, we again employed imaging flow cytometry, performing a single experiment using Draq5 nuclear dye and an anti-AGAT-1 antibody to stain cell suspensions prepared from WT planarians. The antibody was custom-produced and has not been extensively validated. The Draq5 and anti-AGAT-1 double positive cells were sequentially gated using unstained sample as control (Figure 8A) The analysis revealed that ∼25–30% of Draq5^+^ cells were positive for AGAT-1. Further, majority of MuNs (16 out of 23 events, 70%) were found to be a part of the AGAT-1^+^ pool of cells suggesting that these cells are likely to represent a pool of late epidermal progenitors (Figure 8B). However, it is important to emphasize that AGAT-1 is not exclusive to MuNs and, therefore, cannot be considered a MuN-specific marker. Additionally, given the characteristics of the antibody used, the above results warrant further verification.

## Discussion

Polyploidization occurs in both development and disease, including cancer, with mechanisms and outcomes varying across contexts. Multinucleated cells, or MuNs, are types of polyploid cells playing crucial roles in processes like development, regeneration, and wound repair across species (Anatskaya and Vinogradov, 2022). Despite their importance, MuNs have remained largely unexplored in planarians. Prior to this study, such cells had been documented in asexual planarians only once, linked to an externally induced abnormality (Lai et al., 2018).

Here, we present the first report on the identification and preliminary characterization of presumed MuNs in wild-type planarians. Conventional flow cytometry cannot resolve MuNs from events exhibiting high nuclear dye signals due to other factors, like cells in DNA synthesis (S) phase. Microscopy, while providing high-resolution imaging and information about the signal location, is limited in capturing the full diversity of cell types in organisms like planarians due to biases introduced by tissue sections, restricted field of views under study and inadequate throughput. Utilizing the imaging flow cytometry platform, combined with the traditional FACS, we developed an innovative method to overcome the limitations of microscopy and conventional flow cytometry, and study the rare planarian MuNs.

Due to methodological limitations, nuclear envelope staining could not be performed. This raises the possibility that the Draq5-stained spots represent brightly labeled nuclear foci rather than distinct nuclei. However, this scenario appears unlikely. First, MuNs consistently displayed notably stronger Draq5 staining than other cells, indicative of higher ploidy rather than merely a different nuclear organization. Second, the Draq5-labeled spots in MuNs generally did not differ in size from the nuclei of other cells. These observations support the identification of MuNs as presumed multinucleated cells.

Cell diameter measurements from IS-generated BF images showed that MuNs are significantly larger than MoNs, while nuclear sizes generally remain consistent across cells of varied nuclearities, as indicated above. This suggested that MuNs are polyploid, with each nucleus containing genetic material comparable to that of diploid MoNs. We therefore propose that planarian MuNs are formed through cell fusion and/or endomitotic events, rather than endocycling, as no nuclear enlargement was observed (Dörnen et al., 2020). This hypothesis is supported by the presence of both odd and even nuclearities in MuNs, as well as by their cell membrane patterns. Additionally, the presence of MuNs in similar proportions under both wild-type regenerating and non-regenerating conditions suggests that they are physiological cell types. Next, our marker gene expression analyses and neoblast ablation studies provided initial insights into the differentiative potential of MuNs in planarians. We proposed that MuNs are likely undifferentiated cells, possibly progenitors, based on several key observations. Firstly, in wild-type worms, FACS population E containing MuNs was majorly composed of undifferentiated cells–with highest expression of the late epidermal progenitor marker *Agat-1* among all populations analyzed (Figure 4C). Secondly, the neoblast elimination via *H2B* KD had the greatest negative impact on FACS population D, where it caused a pronounced reduction of cell percentage, while population E was less affected (Figure 6D). Lastly, a direct visual inspection of MuNs unequivocally demonstrated that their proportion in the total cell pool marginally declined in *H2B* KD worms (Figure 6B). Based on the data regarding the specific cell types under *H2B* KD conditions at day 5 post-RNAi, one would expect clearance of MuNs if they were neoblasts. In contrast, if these cells were differentiated, no effect would be anticipated (Solana et al., 2012). The observed pattern of their limited decline instead indicated that MuNs could be progenitors. In this context, an important question arose whether MuNs represent a single lineage and, if so, whether they are of epidermal identity.

Population E, comprising MuNs, harbors a substantial proportion of *Agat-1* ^+^ cells (Figure 4C), which represent late epidermal progenitors (Eisenhoffer et al., 2008). The planarian epidermal lineage is well-characterized and originates from zeta neoblasts within the mesenchyme (van Wolfswinkel et al., 2014; Wurtzel et al., 2017). The marker genes *NB21.11e* and *Agat-1* are associated with early and late epidermal progenitors, respectively, and according to a previous study, their expression diminished to varying degrees upon *H2B* knockdown, with *NB21.11e* decreasing more than *Agat-1*. Moreover, while *NB21.11e* became undetectable at day 10 post-RNAi, cells expressing *Agat-1* persisted throughout the follow-up, as long as 20 days (Solana et al., 2012). Likewise, following neoblast ablation through lethal irradiation, *NB21.11e* ^+^ early epidermal progenitors were more susceptible to irradiation-induced damage than *Agat-1*-positive late epidermal progenitor cells (Eisenhoffer et al., 2008). The extent of reduction of *Agat-1* expression level in *H2B* KD worms in this study (Figure 6A) parallels with the limited reduction in the abundance of MuNs (Figure 6B). Therefore, it was tentative to speculate that MuNs might represent late epidermal progenitors. Consequently, we next silenced *Agat-1*, which resulted in a significant decrease in the proportion of MuNs. Subsequently, using a custom anti-AGAT-1 antibody, we demonstrated that the majority of MuNs are positive for this marker. However, AGAT-1 was not specific to MuNs, as approximately 20%–30% of all cells in the analyzed pool were AGAT-1^+^. Moreover, not all MuNs were AGAT-1^+^ (Figure 8A). Thus, while these findings support our current notion that most MuNs are likely late epidermal progenitors, gene expression profiling of MuNs will be required to elucidate their true identity.

Currently, there are no effective methods available for the selective analyses of MuNs. This is due to the fact that in flow cytometry they do not form a distinctive population that could be selectively gated for cell sorting. Even with the latest imaging-based sorting instruments, little additional insight is likely to be gained unless precise automated approaches exist to identify MuNs in real time rather than post-acquisition. In addition, the application of *in situ* hybridization techniques, widely used in planarian research, is challenging, as these cells are rare, which makes it difficult to ensure reproducibility and statistical robustness. One of the most effective approaches for definitively characterizing the identity and function of MuNs is single-cell RNA sequencing (scRNA-Seq). This approach is particularly well-suited to determine whether MuN-specific markers exist, or whether these cells share markers with their lineage and are not molecularly distinct. A first step in this direction would be to enrich MuNs by cell sorting. Next, the resultant population could be subjected to high-throughput scRNA-Seq, using leading microfluidic methods that allow the analysis of tens of thousands of cells. Such an approach would increase the likelihood of characterizing MuNs even in the absence of selective sorting. To achieve this, it is imperative to establish a comprehensive experimental and analytical framework. A critical initial step involves the extensive validation of the anti-AGAT-1 antibody and its application in cell sorting to obtain populations enriched in MuNs. It is also necessary to collect sufficient material, as MuNs are rare, and to apply gentle sorting procedures to maximize the survival of these cells, which are likely to be more susceptible to disintegration than more compact MoNs. Subsequently, data analysis methodologies for scRNA-Seq will need to be refined. Current approaches primarily focus on filtering out cells that do not meet criteria for being typical single cells, which may inadvertently exclude MuNs. This issue requires careful adjustment to ensure that MuNs are accurately identified and analyzed within the broader dataset. Another promising approach for further characterization of MuNs involves systematic RNAi disruptions of the epidermal lineage and analysis of MuNs using imaging cytometry, with particular attention to their morphological characteristics and changes in abundance under different conditions.

Naturally occurring polyploid cells, found, for example, in *Drosophila* and mammalian tissues, are typically post-mitotic and rarely proliferative but exhibit high ploidy plasticity to meet functional demands (Windmueller et al., 2020). They utilize this plasticity in regeneration by functioning either as reservoirs of diploid cells or as immediate effectors in wound healing. The first mechanism occurs during starvation in *Drosophila*, when intestinal stem cells are depleted and rapidly reappear upon feeding, largely through depolyploidization of 4n enterocyte lineage cells (Lucchetta and Ohlstein, 2017). Similarly, mammalian hepatocytes reduce ploidy under proliferative stimuli, such as during post-injury regeneration or during metabolic stress (Duncan et al., 2010). The second mechanism takes place elsewhere in *Drosophila*, where polyploidization mitigates tissue loss, from epidermal wounds to hindgut damage, via cell fusion and repeated endocycles (Losick et al., 2013). However, it is also important to note that multinucleated cells are present under physiological conditions in various organisms, where they arise through the formation of syncytia. In *Caenorhabditis elegans* and *Drosophila*, syncytia play a crucial role not only in development but also in the physiology of the adult organism (Alper and Podbilewicz, 2008; Kloc et al., 2024). Thus, further studies on the mechanisms underlying the formation of planarian MuNs described in this work are necessary for a proper understanding of their functions and significance in an evolutionary context.

In conclusion, the identification of MuNs in *S. mediterranea* introduces a novel dimension to the study of planarian cellular biology. Given their probable epidermal progenitor cell status, investigating their potential involvement in regenerative processes constitutes a promising direction for future research. Additionally, the techniques described here may inspire researchers seeking to uncover rare unexplored cell types in other model organisms.

## Data Availability

The raw data supporting the conclusions of this article will be made available by the authors, without undue reservation.

## Supplementary material

SUPPLEMENTARY FIGURE S1
**(A)** IS image gallery of exemplar MuNs obtained using alternative Hoechst 33342 nuclear staining. Scale: 7 μm; magnification: 40×. **(B)** Calcein AM versus Hoechst 33342 intensity plot showing 4 gated population (I-IV), with population II containing MuNs (green dots).

SUPPLEMENTARY FIGURE S2 IS image galleries of FACS sorted populations **(A–D)**. Scale: 7 μm; magnification: 40×.

SUPPLEMENTARY FIGURE S3
**(A)** Specific population gating strategy skipping the singlets gating step for FACS sorting, to capture larger objects. Encircled region in the plot corresponding to Step 4 shows the co-localization of population E1 (skipping singlets gating) and population E (strategy including singlets gating). **(B)** IS image gallery of exemplar MuNs from FACS sorted population E1. Scale: 7 μm; magnification: 40×.
