# Supplementary material for: Identification and characterization of planarian multinucleated cells in Schmidtea mediterranea using imaging flow cytometry
Source: Front Cell Dev Biol. 2025 Nov 28;13:1611516. doi: 10.3389/fcell.2025.1611516 (PMC12698644; doi:10.3389/fcell.2025.1611516)
Supplement: Supplementary file 2 [file Table1.docx]

Supplementary table 1 (Table S1): List of primer sequences used for qPCR amplifications and synthesis of dsRNAs targeted against *H2B* and *Agat-1* genes respectively.

| **Name** | **Primer sequence** |
| --- | --- |
| *H2B_forward_T7* | 5’ TAATACGACTCACTATAGGGTCTGTTAAGAAGATTTCAAAGG 3’ |
| *H2B_reverse_T7* | 5’ TAATACGACTCACTATAGGGTCCTGTGTATTTTGTAACAGC 3’ |
| *Agat-1_forward_T7* | 5’ TAATACGACTCACTATAGGGAAAGTCGTCCATCCAGAACC 3’ |
| *Agat-1_reverse_T7* | 5’ TAATACGACTCACTATAGGGCTCCAAGTCATGGTGGACTC 3' |
| *H2B_forward_qPCR* | 5’ AGTTGAACGGCCCTCTTTAG 3’ |
| *H2B_reverse_qPCR* | 5’ ACGTACTTCAACGACGTTTT 3’ |
| *NB21.11e_forward_qPCR* | 5’ AAAGTCTCCCGCCAAATCAA 3’ |
| *NB21.11e_reverse_qPCR* | 5’ CGCAATCTTTGTCGAGCTTC 3’ |
| *Smed1_forward_qPCR* | 5' AAACGTGAGCCTAGAGAACG 3' |
| *Smed1_reverse_qPCR* | 5' GACCACGAATCGTAATCGGT 3' |
| *Agat-1_forward_qPCR* | 5' AGAGGAACCAGTTTTCGACG 3' |
| *Agat-1_reverse_qPCR* | 5' AGGTGTGAAAAGTGTCGTGT 3' |
| *GST-1_forward_qPCR* | 5' AATGGCCAGAAGTGAAACCA 3' |
| *GST-1_reverse_qPCR* | 5' AACCCATGTTTTCGTGCAAC 3' |
| *PC2_forward_qPCR* | 5’ GCATTTGTGGTGTTGGAGTG 3’ |
| *PC2_reverse_qPCR* | 5’ TCATTTCTCGGCCCATCTAC 3’ |
| *H.55.12e_forward_qPCR* | 5' TTCCTACAGCCACTTGAGCGAC 3' |
| *H.55.12e_reverse_qPCR* | 5' GTCGGTGGTTATTTTGCG 3' |
| *Ef2_forward_qPCR* | 5’ TGCTGGTGACACTTTGCTTC 3’ |
| *Ef2_reverse_qPCR* | 5’ CATCACCAAGTGTCCGTTTG 3’ |
