# Supplementary figures and images for: Identification and characterization of planarian multinucleated cells in Schmidtea mediterranea using imaging flow cytometry
Source: Front Cell Dev Biol. 2025 Nov 28;13:1611516. doi: 10.3389/fcell.2025.1611516 (PMC12698644; doi:10.3389/fcell.2025.1611516)

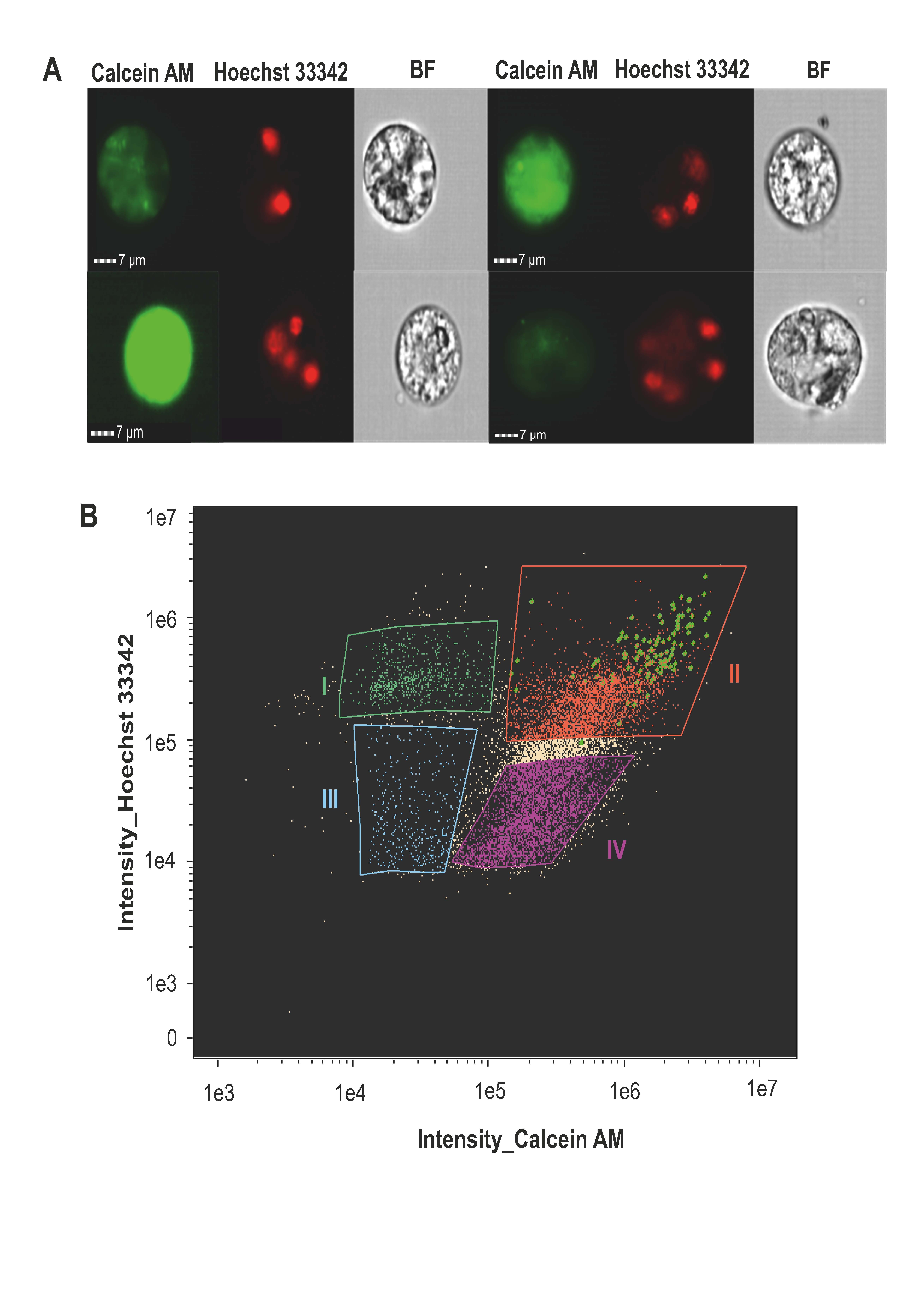

Supplement: Supplementary file 3 [file Image1.jpeg]

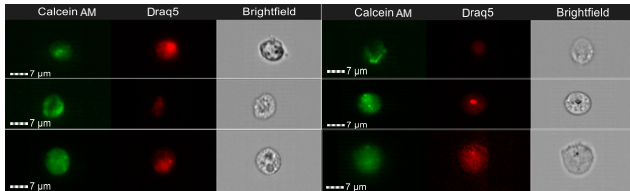

**Population A**

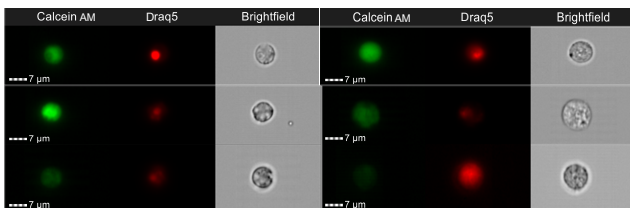

**Population B**

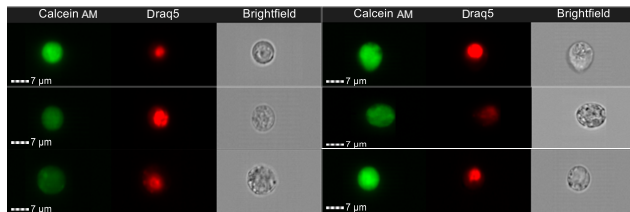

**Population C**

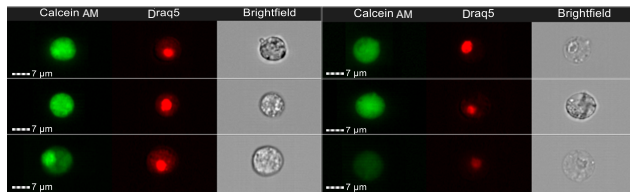

**Population D**

Supplement: Supplementary file 4 [file Image2.pdf]

**A**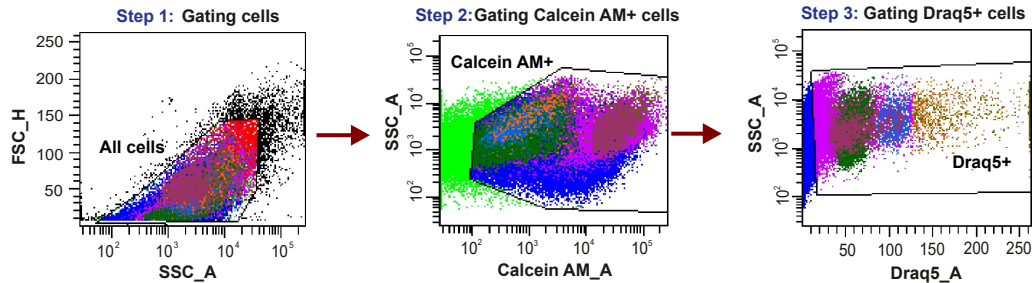**B**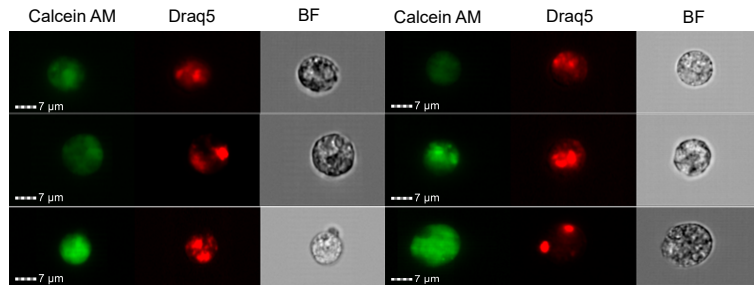

**Step 4: Gating different cell populations based on Calcein AM and Draq5 staining intensities**

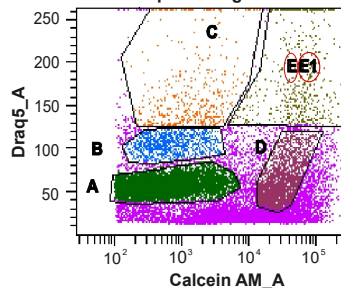

Supplement: Supplementary file 5 [file Image3.pdf]
